# Long-Term Monitoring of Physical Behavior Reveals Different Cardiac Responses to Physical Activity among Subjects with and without Chronic Neck Pain

**DOI:** 10.1155/2015/907482

**Published:** 2015-10-18

**Authors:** David M. Hallman, Svend Erik Mathiassen, Eugene Lyskov

**Affiliations:** Department of Occupational and Public Health Sciences, Centre for Musculoskeletal Research, University of Gävle, Kungsbäcksvägen 47, 801 76 Gävle, Sweden

## Abstract

*Background*. We determined the extent to which heart rate variability (HRV) responses to daily physical activity differ between subjects with and without chronic neck pain. *Method*. Twenty-nine subjects (13 women) with chronic neck pain and 27 age- and gender-matched healthy controls participated. Physical activity (accelerometry), HRV (heart rate monitor), and spatial location (Global Positioning System (GPS)) were recorded for 74 hours. GPS data were combined with a diary to identify periods of work and of leisure at home and elsewhere. Time- and frequency-domain HRV indices were calculated and stratified by period and activity type (lying/sitting, standing, or walking). ANCOVAs with multiple adjustments were used to disclose possible group differences in HRV. *Results*. The pain group showed a reduced HRV response to physical activity compared with controls (*p* = .001), according to the sympathetic-baroreceptor HRV index (LF/HF, ratio between low- and high-frequency power), even after adjustment for leisure time physical activity, work stress, sleep quality, mental health, and aerobic capacity (*p* = .02). The parasympathetic response to physical activity did not differ between groups. *Conclusions*. Relying on long-term monitoring of physical behavior and heart rate variability, we found an aberrant sympathetic-baroreceptor response to daily physical activity among subjects with chronic neck pain.

## 1. Introduction

Chronic pain in the neck region is a common condition [1], particularly in the working population [2]. Long-term, continuous monitoring of behavioral and physiological responses in daily living opens new opportunities for gaining knowledge on the pathophysiology, prevention, and treatment of these pain conditions.

Research suggests that the autonomic nervous system (ANS) is involved in the development and persistence of chronic muscle pain at both central and peripheral levels [3–5], as well as in adaptive responses to acute experimental pain [6].

Heart rate variability (HRV) is a valid and reliable biomarker of autonomic regulation, including parasympathetic and sympathetic-baroreceptor influences on cardiac modulation [7–10]. Autonomic activity, as assessed through HRV indices in time and frequency domains, differs between subjects with and without chronic neck pain during controlled laboratory rest [11] and during sleep [12, 13]. This reflects a reduced basal parasympathetic activity in subjects with pain, corroborating studies using heart rate and blood pressure as measures of ANS activity at rest [14, 15].

Even the HRV response to physical work appears to be affected in pain conditions. In a recent study [11], chronic neck pain was associated with attenuated low-frequency (LF) spectral power during submaximal isometric handgrip, while high-frequency (HF) spectral power was similar for the pain and control groups. Similarly, Shiro et al. [16] found the LF/HF ratio to be increased in healthy subjects during maximal isometric contractions of the trapezius muscles, while no change in LF/HF was found in subjects with neck pain, suggesting an aberrant sympathetic-baroreceptor response to isometric exercise in pain, and parasympathetic withdrawal is normal. The latter was confirmed by Elcadi et al. during sustained shoulder elevation [17]. These autonomic responses are clinically important as they may contribute to altered pain processing [4, 18], decreased tolerance to physical loads [19], and poor cardiovascular prognosis [20, 21]. Thus, maladaptive autonomic responses to physical work may be involved in maintaining chronic neck pain.

While pain and control groups differ in HRV response to controlled physical work, no study has, to our knowledge, investigated the extent to which HRV is altered in response to naturally occurring physical activity in people with chronic neck pain, with due consideration to essential confounders, such as mental and physical health [22], sleep quality [23], and work stress [24]. Notably, chronic pain is often associated with reduced levels of physical activity [25], which is, in turn, associated with reduced HRV [26–28].

Studies of physical activity require accurate and precise measurement methods, preferably based on objective devices such as accelerometers, as self-reported measures of physical activity are less reliable, prone to bias [29], and operate at a level of resolution which may not be sufficient to disclose associations between, for instance, the temporal structure of physical activity and health outcomes. In this context, it is important to discriminate between physical activity practiced at work and during leisure, which may differ markedly in both structure and effects. For instance, studies have found that leisure time physical activity is beneficial for cardiovascular health [30], including an enhanced autonomic function [27], while occupational physical activity may be even detrimental [31, 32]. Work and leisure periods can be separated by means of self-reports, which may, however, be both time-consuming and disturbing for the participant, while providing less precise data compared to objective methods [33]. Using information from the Global Positioning System (GPS) is an established tool for objective assessment of time series of geographical data [34, 35], and it does allow for a detailed separation of periods of work and leisure. The present study aimed at determining the extent to which HRV responses to different types of physical activity differ between subjects with chronic neck pain and healthy controls and at investigating whether these HRV responses differ between work and leisure, as identified by GPS complimented with diaries. We hypothesize that chronic neck pain will be associated with an aberrant HRV response to daily physical activity, as compared with no pain.

## 2. Methods

### 2.1. Subjects

Twenty-nine workers (13 women, 16 men; mean age 41 (SD = 10) years) with chronic neck pain and 27 age- and gender-matched healthy workers without a recent history of pain participated. Subjects were recruited through advertisement at a large industrial plant in Sweden (>5000 employees at site) belonging to a global steel manufacturing company, in cooperation with ergonomists and health care specialists working at the company.

First, eligibility was evaluated using interviews and questionnaires followed by a physical examination. Inclusion in the pain group required nontraumatic chronic pain (>6 months) localized to the neck-shoulder region (i.e., primarily the neck and/or the regions corresponding to the trapezius muscles according to a pain drawing). Controls were included only if they were reported to be healthy and asymptomatic. Both pain and control subjects had to be between 20 and 59 years of age and to work at least 75% of full-time work. Both males and females were allowed into the study. Exclusion criteria included regular use of medication that could affect cardiovascular function or pain perception (e.g., antidepressants, beta-blockers, and anti-inflammatory drugs). Individuals were also excluded if they reported comorbidity with other disorders known to affect physical activity, autonomic regulation, or pain processing (e.g., diabetes, depression, and cardiovascular diseases), drug abuse, pain of traumatic origin (e.g., whiplash associated disorders), or neuropathic pain conditions. Workers were also excluded if reporting sick leave more than 2 weeks within the past three months.

Eligible subjects with and without pain were examined by a specialized physiotherapist [36]. Subjects were classified as having chronic neck pain, corresponding to the International Classification of Diseases (ICD-10) code M 79.1, if they reported chronic pain from the neck-shoulder region, muscle stiffness, and tenderness at palpation without restricted range of motion of the neck during the examination [36–38]. All subjects were given information about the study prior to participation and provided written informed consent. The study was approved by the regional ethical review board in Uppsala, Sweden, and was conducted according to the Declaration of Helsinki.

### 2.2. Procedure

Data were collected from May 2011 to June 2012, although no data were collected from November to April to minimize seasonal effects on physical activity. Shortly after being recruited for the study, subjects filled in a battery of questionnaires (below) and went through a long-term recording of objectively measured physical activity, HRV, location by GPS, and self-reported symptoms [12]. With few exceptions, the ambulatory measurement period started at the beginning of a regular week and lasted for up to seven days. An accelerometer for assessment of physical activity was worn for seven days, while a heart rate monitor and a smartphone, containing an electronic diary and GPS software, were worn by the subjects for approximately 72 hours (i.e., the first three days of the seven-day recording), typically representing three full workdays of daytime work. For all of these measures, only data from the first 72 hours were analyzed in the present study. Subjects were equipped with the assessment devices at their work place. The devices were only removed during a shower or a bath and replaced shortly after that. Subjects were instructed to wear the smartphone during all waking hours and to rate their perceived stress level when prompted by an auditory signal (see below). They were instructed to perform their regular activities and were advised to contact the examiner if they had any complaints caused by the data collection.

### 2.3. Assessment of Work and Leisure Periods

GPS coordinates were sampled at 0.2 Hz using the freely available software Map WM (http://www.mapwm.com/) installed on a Smartphone (HTC* HD2*) with a Windows operative system. The GPS coordinates combined with self-reported periods of work, leisure, and sleep, were used to identify periods of work and leisure time, the latter classified as either “at home” or “elsewhere.” Working hours were identified solely from the diary reports, and leisure time, whether “at home” or “elsewhere,” was recognized only if the diary indicated leisure time. The “at home” location was identified as the spatial region within 50 meters from the median GPS position during sleep and “elsewhere” was defined as anywhere outside this “at home” region. Sleep periods were not considered in the present study. Thus, all temporal data (i.e., physical activity, HRV, and stress ratings) were partitioned according to whether it occurred during “work,” leisure “at home,” or leisure “elsewhere.”

### 2.4. Objectively Measured Physical Activity

Physical activity was objectively measured using a single triaxial accelerometer (ActivPAL; PAL Technologies Ltd., Glasgow, UK) attached to the thigh using self-adhesive tape, producing data at 20 Hz. The device has shown good validity and reliability in detecting different types and intensities of physical activity in daily life [39–41]. Time spent walking, standing, and sitting/lying, number of steps, and cadence (steps/minute) were calculated offline using the commercial software accompanying the accelerometers. For each of these activities, the average metabolic equivalent (MET/hour) was estimated [42] as a measure of energy expenditure (i.e.,* sitting/lying *= 1.25 METs;* standing* = 1.4 METs;* stepping* 120 steps/minute = 4 METs; the increase in walking energy expenditure was estimated to be 0.22 METs for every increment of 10 steps/minute from standing, i.e., 0 steps/minute).

### 2.5. Heart Rate Variability

Interbeat electrocardiogram intervals (IBIs) were collected using a heart rate monitor (Firstbeat Bodyguard; Firstbeat Technologies Ltd., Jyväskylä, Finland) attached using preglued Ag/AgC1 electrodes (Biopac Systems Inc., USA) on cleansed skin. IBI time series were first processed and analysed using Firstbeat HEALTH (version 3.1.1.0, Firstbeat Technologies Ltd., Jyväskylä, Finland), using procedures for automatic data editing and short-term Fourier transform filtering described by Saalasti [43]. Only periods free from artefacts due to, for example, noise, ectopic beats, or nonwear time, were analysed. On average, one recording included 97.4% (SD 3.7%) acceptable data. HRV was analysed according to Task Force [44] in the time domain (i.e., IBI and the square root of the mean squared successive differences of IBIs RMSSD) and the frequency domain (i.e., the ratio between low-frequency (LF 0.04–0.15 Hz) and high-frequency (HF 0.15–0.4 Hz) spectral power (ms^2^), LF/HF). RMSSD was used as a measure of parasympathetic (vagal) activity [7, 9], while LF/HF was used as a measure of sympathetic-baroreceptor activity [44, 45].

### 2.6. Self-Reported Neck Pain

Pain localization was assessed using a modified pain drawing [46]. The average perceived pain intensity in the neck region during the previous “six months” and “seven days” was rated using the Borg CR10 scale [47]. The response scale ranges from 0 (“nothing at all”) to 10 (“extremely strong”).

### 2.7. Assessment of Potential Confounders

Gender, age, weight, height, and type of work (office or production) were assessed by self-reports.

The short form health survey (SF-36) was used to assess health-related functions and quality of life [48]. The mental health component (one out of eight dimensions in SF-36) rated on a 0–100 scale was used in the present study, whereby a higher score reflects better health.

The Karolinska Sleep Questionnaire (KSQ) [49] was used to assess sleep quality based on four items: difficulty falling asleep, repeated awakenings, premature awakening, and disturbed sleep. Subjects rated their experiences over the past six months using a response scale from 1 (always) to 6 (never). The four sleep quality items were added up to create a sleep quality index ranging from 0 to 24, whereby higher values indicated better sleep.

The intensity of current symptoms (pain, stress, and fatigue) was assessed in a custom-made electronic diary, installed on the smartphone. Thus, the intensity of “current” stress was assessed using the CR10 scale [47] 30 minutes after waking up in the morning, every second hour from 09:00 to 17:00, at 20:00, and just before going to bed. An auditory reminder was repeated three times at ten-minute intervals in case a rating was missed.

Aerobic capacity, VO_2max_, was assessed using a submaximal cycle ergometer test according to Åstrand and Rhyming [50].

### 2.8. Further Processing of Heart Rate Variability (HRV)

All data obtained from the 72-hour recording, including the objective measurements (GPS coordinates, IBI, frequency HRV values (see below), physical activity types, and METs) and the stress ratings were imported to the Spike2 software (version, 7.03, Cambridge Electronic Design) for visual data inspection and further data processing.

Each HRV index was assessed for periods classified as sitting/lying, standing, and walking, respectively, for each of the three locations of work, leisure at home, and leisure elsewhere. Series of IBIs and successive differences of IBIs were concatenated within each activity category (periods containing less than 3 IBIs were excluded). For each activity type, we calculated the average IBIs, RMSSD (average of 5 min RMSSD epochs), and LF/HF (averages of 1 min LF/HF epochs).

In total, 49 subjects (pain, *n* = 25; control, *n* = 24) with acceptable data on GPS, physical activity, and HRV were included in the statistical analyses. The analysis of LF/HF included only 42 subjects (pain, *n* = 20; control *n* = 22), mainly due to a lack of standing periods exceeding 1 min in leisure “elsewhere” for some subjects.

### 2.9. Statistical Analyses

Descriptive data are presented as frequencies or as mean with standard deviation (SD) between subjects. Chi^2^ tests were used to test for differences between pain and control groups in the distribution of gender and work type (office versus production). *t*-tests for independent samples were used to test for group differences (pain versus control) in age, BMI, pain intensity, energy expenditure (METs), work stress, mental health, and sleep quality, as well as the duration of work and leisure periods.

Repeated measures ANOVA models were constructed to analyze HRV indices using* activity type* (3 levels: sit/lie, stand, and walk) and* location* (3 levels: work, leisure “at home,” and leisure “elsewhere”) as within-subject factors and* group* (2 levels: pain, control) and* work type* (2 levels: office, production) as between-subjects factors. In a second step, we included METs in leisure time “elsewhere” as a covariate to investigate the potential association between the extent of physical activity during leisure time, HRV, and pain.

In addition, the same mixed ANOVAs were expanded with a step-wise inclusion of covariates (ANCOVA) in the following order: age, gender, BMI, METs (leisure “elsewhere”), work stress, mental health, sleep quality, and VO_2max_, all of which were selected based on previous reports of their relationship with autonomic function and pain. Covariates were excluded from the model if they showed *p* values larger than .10 for either their main effect on HRV or their interaction with* activity type*. All statistical analyses were performed using SPSS, version 22. *p* values less than .05 were considered to indicate significant effects.

## 3. Results

Table 1 shows descriptive variables in the pain and control groups. No group differences were observed for age, gender, work type, body mass index (BMI), or aerobic capacity (VO_2max_). In the pain group, the self-reported duration of neck pain was, on average, 10.1 (SD 8.5) years, and the intensity of neck pain corresponded to “somewhat strong” according to the CR10 scale, for both the past six months and the past seven days. The average number of work days with acceptable recordings of GPS, HRV, and accelerometry was similar for the two groups. Also, there were no significant differences between the groups in total measured time at work or leisure “at home” and “elsewhere” (all *p* > .45). The pain group reported significantly higher perceived stress at work than the controls, although stress levels were overall quite low. Perceived mental health and sleep quality were reduced in the pain group compared with controls, although without reaching significance (*p* > .05).

**Figure 1:**
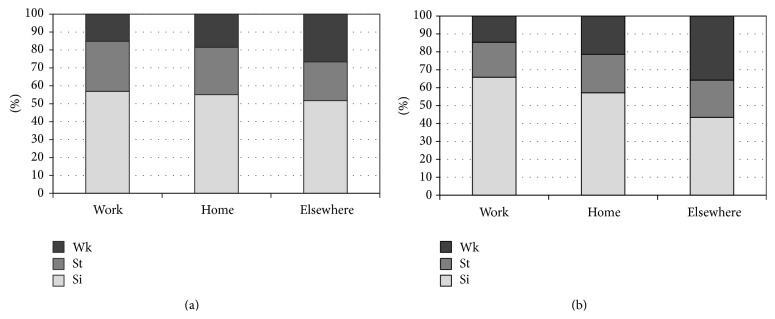
Time spent in physical activity (Si, sit/lie; St, stand; Wk, walk) in pain (a) and control (b) groups. Percentage of analyzed time is shown on the *y*-axis, and the spatial locations are shown on the *x*-axis.

For physical activity in terms of estimated accelerometry-based MET values, the pain group had significantly lower METs during leisure “elsewhere” than the controls, while no difference was found at work or leisure “at home.” Figure 1 shows the proportion of time spent in different physical activities across locations in both groups. In comparison with work, leisure time “elsewhere” was characterized by an increased proportion of time spent in walking and reduced time in sitting/lying in the control group, while this increase in physical activity during leisure did not occur to the same extent in the pain group.

### 3.1. Effect of Activity Type and Location on HRV

**Figure 2:**
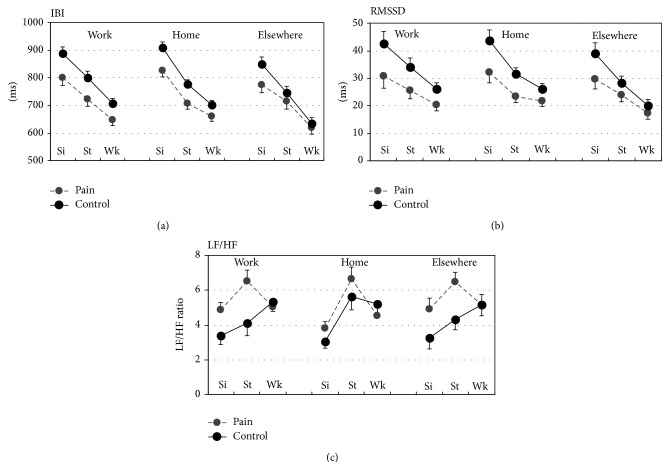
Heart rate variability (IBI, interbeat interval; RMSSD, root mean squared successive differences between IBIs; LF/HF, ratio between low- and high-frequency spectral power) determined for different physical activities (Si, sit/lie; St, stand; Wk, walk) during work and leisure (home and elsewhere) for the pain and control groups.

Significant effects of* activity type* were found for HRV (Tables 2 and 3). IBI and RMSSD decreased with increasing physical activity (i.e., sit/lie, stand, and walk), while LF/HF increased from sit/lie to stand. IBI and RMSSD differed depending on* location* (Table 3, Figure 2); both were reduced for leisure “elsewhere” compared to work and leisure “at home.” There was no significant effect of* location* on LF/HF, and there were no significant interactions between* activity type* and* location* for any of the HRV indices.

### 3.2. Differences in HRV between Pain and Control Groups

Main effects of* group* (pain versus control) were found on HRV (Table 3), with reduced IBI and RMSSD, and increased LF/HF in the pain group compared with controls. However, only the IBI difference reached significance. We found a significant interaction (*activity type* ×* group*) for LF/HF (Table 3), with an attenuated LF/HF response to walking (i.e., compared to sitting/lying or standing) in the pain group compared with controls (Figure 2). Post hoc tests showed that this interaction was significant for work (*F*(2,90) = 7.6; *p* = .001) and leisure “at home” (*F*(2,90) = 5.1; *p* = .03), while it did not reach significance for leisure “elsewhere” (*F*(2,76) = 2.3; *p* = .11). The three-way interaction (*activity type* ×* location* ×* group*) was not significant for any HRV index.

Additional ANCOVA models (Table 4) for LF/HF with step-wise adjustments for multiple covariates showed that the interaction effect (*activity type* ×* group*) remained significant after adjustments; only age and gender came out as significant covariates in the model. We also accounted for a possible influence of IBI on LF/HF by regressing IBI against LF/HF for each activity type and by rerunning the ANOVA using the residuals from the regression models as dependent variables. The interaction between* activity type* and* group* was still significant (*F*(2,90) = 6.2; *p* = .003).

### 3.3. Association between Leisure Time Physical Activity and HRV

The difference between* activity types* in IBI (*F*(2,88) = 4.3; *p* = .03) and RMSSD (*F*(2,88) = 9.7; *p* = .002) depended on the level of leisure time physical activity (METs “elsewhere”), with a larger decline in HRV in response to walking among those subjects having a larger estimated MET value. For RMSSD, this interaction was also significant for work (*F*(2,88) = 8.2; *p* = .004). Also, the difference between* locations* in RMSSD (*F*(2,88) = 3.6; *p* = .03), but not in IBI (*p* = .39) or LF/HF (*p* = .81), depended on the metabolic level of leisure time physical activity, with a higher leisure MET being associated with enhanced HRV for work, but not for leisure.

When adjusting for METs in leisure “elsewhere” as a covariate in the ANCOVA, the differences in HRV between the three activity types and locations turned substantially less conclusive than what appeared in the ANOVA without adjustment for METs (Table 3), that is,* activity* main effect: IBI, *F*(2,88) = 2.64; *p* = .08; RMSSD, *F*(2,88) = 3.29; *p* = .07; LF/HF, *F*(2,74) = 2.27; *p* = .11;* location* main effect: IBI, *F*(2,88) = 0.42; *p* = .66; RMSSD, *F*(2,88) = 2.61; *p* = .08; LF/HF, *F*(2,74) = 0.24; *p* = .78.

Also, with inclusion of MET “elsewhere” in the model, the group differences (pain versus control) in HRV were reduced, and for IBI it did no longer reach significance (IBI, *F*(1,44) = 2.91; *p* = .10; RMSSD; *F*(1,44) = 0.79; *p* = .38; LF/HF, *F*(1,37) = 2.07; *p* = .20). The interaction between* activity type* and* group* remained significant for LF/HF even after adjustment for physical activity (MET) in leisure time “elsewhere” (Table 4). This means that group difference in the LF/HF response to physical activity was not explained by a reduced level of leisure time physical activity in the pain group.

## 4. Discussion

The present study investigated the extent to which autonomic responses (measured through HRV) during physical activity at work and during leisure differ between subjects with and without chronic neck pain. We found that subjects with chronic neck pain had a reduced sympathetic-baroreceptor component of HRV in response to physical activity, as compared with controls, even when accounting for a wide range of potential confounders.

### 4.1. Autonomic Response to Sitting, Standing, and Walking

As expected, IBI (i.e., reciprocal heart rate) and RMSSD (Figure 2) decreased between lying/sitting and standing and further between standing and walking, reflecting an attenuated parasympathetic (vagal) cardiac modulation with an increase in physical activity. This activity-induced attenuation of parasympathetic activity was not significantly different between the pain and control groups, which is in agreement with laboratory studies assessing parasympathetic HRV indices during controlled physical exercise, that is, isometric contractions; [11, 17].

We found, however, that subjects with chronic neck pain had an aberrant LF/HF response to daily physical activity compared with healthy controls. That is, the control group showed an increased LF/HF when changing from a sedentary position to standing and walking, which corroborates previous reports [51, 52], while the pain group showed a reduced LF/HF response from standing and sitting/lying to walking (Figure 2, Tables 2–4). This indicates a reduced sympathetic-baroreceptor modulation of the heart in response to physical activity among the subjects with chronic pain. This novel finding from a field study of daily activities corroborates laboratory studies showing attenuated LF and LF/HF components during isometric contractions among people with neck pain compared with healthy controls [11, 16]. This is also consistent with two studies showing attenuated arterial blood pressure responses during static [14] and dynamic exercise [53] in people with neck pain compared to controls. In the current study, we even accounted for a wide range of important covariates, such as leisure time physical activity, work stress, mental health, sleep quality, and aerobic capacity. In addition, we adjusted the HRV indices for mean IBI, as previously recommended [54], and found that the LH/HF response remained significantly different between the pain and control groups after this adjustment.

The group difference in LF/HF response to physical activity suggests an aberrant sympathetic-baroreceptor function in subjects with chronic neck pain, according to studies indicating that LF/HF is a sensitive measure of sympathetic-baroreceptor activity during experimentally induced pain [6]. The onset and continuation of a physical activity bout are accompanied by a reduced IBI, as confirmed by our data, which is due to a shift in autonomic balance favoring sympathetic over parasympathetic predominance [55]. During physical activity, the arterial baroreflex regulates arterial blood pressure via changes in sympathetic and parasympathetic cardiac modulation [21], as reflected in LF and HF indices of HRV [56]. The interplay between the baroreflex and the ANS is, in turn, under the influence of central networks [57] involving, for instance, the anterior cingulate and insular cortices and the thalamus, as well as structures in the brain stem (periaqueductal grey, medulla), which are also engaged in central pain processing and descending pain inhibition [58, 59]. Thus, baroreceptor regulation of blood pressure during physical activity is associated with adaptive hypoalgesia, which may be inhibited in conditions of chronic pain [4, 18, 60, 61], including neck pain [62].

Thus, our findings point to the involvement of a central dysregulation in chronic neck pain, including aberrant interactions between the ANS, baroreflex, and central pain processing mechanisms. We suggest future studies to investigate this further in experimental and prospective designs.

### 4.2. Association between Leisure Time Physical Activity and Parasympathetic Activity

We found that the subjects with chronic neck pain were less physically active (i.e., having lower estimated METs) than the controls, particularly during leisure time. This corroborates previous findings from our research group [12, 13]. However, the present study is, to our knowledge, unique in showing that “inactivity” among subjects with pain does not occur during work or at home, but only in other geographical locations (i.e., leisure “elsewhere”). We also found an increased heart rate (lower IBI) and reduced parasympathetic activity (RMSSD) for leisure “elsewhere” compared to leisure “at home,” which most likely reflects an increased intensity of physical activity “elsewhere.” In addition, there was a significant interaction between physical activity level (i.e., estimated MET) and location on RMSSD, which indicates an enhanced parasympathetic cardiac modulation during work, but not during leisure, among those being more physically active in their leisure time. These findings may encourage future interventions to stimulate leisure time physical activity in chronic neck pain populations and to evaluate intervention effects on HRV and pain.

The subjects with chronic neck pain had shorter IBIs and a trend towards reduced RMSSD compared with the controls (Tables 2 and 3), particularly when sitting or lying (Figure 2). We have previously demonstrated a reduced basal parasympathetic activity among people with chronic neck pain in comparison with asymptomatic controls, as assessed using HRV indices during controlled rest or during sleep [11, 13]. However, the group differences in IBI and RMSSD observed in the present sample were less clear when adjusting for leisure time physical activity “elsewhere” in the ANCOVA models. Nevertheless, the observed association between physical activity levels and HRV may have mechanistic implications with respect to the onset and persistence of chronic neck pain. Parasympathetic activation appears to be involved in the inhibition of inflammatory processes, that is, via activation of the cholinergic anti-inflammatory pathway [63]. Thus, resting HRV is negatively associated with systemic levels of proinflammatory cytokines [64, 65]. Based on animal models, proinflammatory markers have been proposed to contribute to work-related muscle pain [66], and some studies show higher concentrations of proinflammatory cytokines among persons with upper-extremity pain [67–69], including work-related neck pain [70]. Thus, we suggest that this possible connection between physical activity, parasympathetic regulation, and inflammation should be further investigated in prospective studies in chronic neck pain populations.

### 4.3. Methodological Discussion

The assessment of HRV combined with long-term continuous recordings using accelerometry and GPS is an obvious strength of the present study. This allowed us to analyze HRV in response to different activity types across different spatial contexts in an approach that was entirely dependent on the access to continuous data for extended periods. Separating leisure time physical activity “elsewhere” from that “at home” led to a more stringent measure of physical activity during leisure, as confirmed by its clear association with HRV. Thus we could appropriately adjust HRV data for objectively measured levels of physical activity. Given the abundance of studies showing that leisure time physical activity is, in its own right, associated with increased HRV (e.g., [26–28]), adjustment for this factor is crucial. A further strength is the recruitment of subjects with and without pain from the same company, while also minimizing confounding due to recruitment bias.

Our study suffers some limitations which need to be acknowledged. We estimated periods of sitting/lying, standing, and walking from the accelerometer recordings, while any further level of detail in discriminating different types of physical activities was not considered feasible. It is possible that further detail, including identification of, for example, periods of swimming, could have led to an even better understanding of factors influencing HRV. Further, the fact that sitting and lying were not separated may also muddle the interpretation of HRV findings, since HRV can change substantially between sitting and supine positions [71]. Our study design does not allow inferences about causal relationships between HRV and neck pain, even if data of HRV, physical activity, and GPS were collected for several days. Such inferences need to be based on experimental designs or prospective studies using repeated sampling of pain characteristics across a longer time span, for example, following the progression of symptoms from an asymptomatic state to chronic pain. Our study also lacks data on pain sensitivity, which precludes us from determining whether changes in HRV were associated with insufficient pain modulation or not. However, we did not consider it feasible to assess pain sensitivity during different activity types across several days. As our assessment methods were selected to be as nonobtrusive as possible, we did not assess ambulatory blood pressure, and, thus, possible relationships between changes in blood pressure and pain could not be tested. Thus, further studies are recommended to resolve these issues. Finally, as data was collected during the brighter part of the year (i.e., primarily in May and June), caution should be paid in generalizing our results to the spring and winter seasons, where patterns of physical activity and inactivity may differ considerably from those during summer.

## 5. Conclusion

We found that subjects with chronic neck pain showed an attenuated LF/HF response to physical activity compared with asymptomatic subjects, even after adjustment for essential confounders. This suggests an aberrant sympathetic-baroreceptor function among subjects with chronic neck pain. In order to further investigate this theory, interventions or experimental protocols manipulating autonomic regulation need to be evaluated with respect to their possible effect on chronic neck pain. Our results were critically dependent on the access to data collected continuously for prolonged periods of time, and so we recommend using long-term monitoring of physical activity, spatial location, and pain even in future prospective investigations of the physiological and behavioral determinants of chronic neck pain.

## Figures and Tables

**Table 1:** Descriptive statistics for subjects with and without chronic neck pain and *p* values for tests of differences between the two groups.

	Group pain	Control	*p*
Males, *n*	13	14	.78
Females, *n*	12	11	
Office work, *n*	17	19	.53
Production work, *n*	8	6	
BMI, mean (SD) kg·m^−2^	24.5 (3.8)	23.8 (3.3)	.66
Age, mean (SD) years	42.2 (9.8)	41.2 (9.3)	.71
Pain intensity^a^ (six months), mean (SD)	4.2 (1.4)	0.4 (0.8)	**<.0001**
Pain intensity^a^ (seven days), mean (SD)	4.0 (1.3)	0.2 (0.4)	**<.0001**
Work stress^b^ (CR10, 0–10), mean (SD)	2.1 (1.0)	1.0 (0.8)	**<.0001**
Mental health (SF-36, 0–100), mean (SD)	75.2 (13.9)	81.6 (11.3)	.08
Sleep quality (KSQ, 0–24), mean (SD)	16.8 (3.9)	18.3 (2.1)	.10
VO_2max_ (O_2_ mL/kg/min), mean (SD)	44.5 (12.1)	42.0 (10.2)	.45
Measurement duration, mean (SD) work days	3.0 (0.7)	3.0 (0.5)	.96
Time at work, mean (SD) hours	26.5 (6.5)	25.5 (5.0)	.53
Time at home, mean (SD) hours	15.6 (5.1)	15.1 (4.8)	.70
Time elsewhere, mean (SD) hours	7.2 (5.1)	7.8 (4.5)	.65
Self-reported sleep, mean (SD) hours/day	6.5 (0.6)	6.4 (0.3)	.44
Energy expenditure, mean (SD) MET/hour			
MET work	1.6 (0.1)	1.6 (0.2)	.78
MET home	1.6 (0.2)	1.7 (0.3)	.36
MET elsewhere	1.8 (0.3)	2.1 (0.4)	**.02**

^a^Pain intensity was reported using the CR10 scale (range 0–10). ^b^Stress ratings from the electronic diary were averaged across all work periods. Continuous variables were tested using independent samples *t*-tests; distributions of gender and work type were tested by chi^2^ tests; significant *p*-values, <.05, are bold faced. BMI: body mass index; MET: metabolic equivalent.

**Table 2:** Mean and standard deviation (SD between subjects) of heart rate variability during different physical activity types (sitting/lying, standing, and walking), averaged across work and leisure periods for the pain (*n* = 25) and control groups (*n* = 24).

HRV index	Group	Sitting/lying	Standing	Walking
		Mean (SD)	Mean (SD)	Mean (SD)
IBI (ms)	Pain	814 (104)	727 (93)	646 (63)
	Control	865 (100)	762 (90)	665 (72)

RMSSD (ms)	Pain	34 (17)	26 (12)	21 (9)
	Control	40 (19)	30 (12)	23 (8)

LF/HF (ratio)	Pain	3.9 (2.4)	5.3 (2.9)	4.5 (2.0)
	Control	3.1 (1.3)	4.0 (1.8)	4.6 (2.1)

HRV, heart rate variability; IBIs, interbeat intervals; RMSSD, root mean square of successive differences between interbeat intervals; LF/HF, ratio between low- and high-frequency spectral power of heart rate variability.

**Table 3:** Results from the repeated measures ANOVAs of heart rate variability indices. *F*-values and *p* values are shown for the effects of activity type, location and group, and their interactions.

Variable	*n*	Main effects						Interaction effects					
		Activity		Location		Group		Activity × group		Location × group		Activity × location × group	
		*F*	*p*	*F*	*p*	*F*	*p*	*F*	*p*	*F*	*p*	*F*	*p*
IBI (ms)	49	**213.6**	**<.0001**	**9.2**	**<.0001**	**5.1**	**.03**	3.2	.07	1.4	.26	0.7	NS
RMSSD (ms)	49	**37.0**	**<.0001**	**4.8**	**.01**	3.8	.06	1.9	.16	0.9	NS	0.3	NS
LF/HF (ms)	42	**18.4**	**<.001**	0.1	NS	2.6	.11	**7.6**	**.001**	2.2	.12	0.2	NS

Note: nonsignificant, NS, *p* > .30; all models are adjusted for type of work.

IBIs, interbeat intervals; RMSSD, root mean square of successive differences between interbeat intervals; LF/HF, ratio between low- and high-frequency spectral power of heart rate variability.

**Table 4:** Results from the ANCOVA analyses of LF/HF HRV, with *p* values for the main effect of group (pain versus control) and the interactions between group, activity type, and location.

Covariates	*n*	Group effect		Activity × group		Location × group	
		*F*	*p*	*F*	*p*	*F*	*p*
Age	42	2.1	.16	**6.1**	**.004**	1.9	.16
Gender	42	2.4	.13	**6.1**	**.004**	1.9	.16
BMI	42	—	—	—	—	—	—
METs, leisure “elsewhere”	42	—	—	—	—	—	—
Work stress, CR10	42	—	—	—	—	—	—
Mental health, SF-36	42	1.5	.23	**4.2**	**.02**	1.1	.93
Sleep quality, KSQ	42	—	—	—	—	—	—
VO_2max_	39	0.09	.77	**6.4**	**.003**	1.7	.20

Note: all ANCOVA models are adjusted for type of work. Stepwise adjustments were made for age, gender, BMI, work stress, mental health sleep quality, and VO_2max_.

— indicates exclusion (*p* > .10) of a covariate from the final ANCOVA model.

BMI, body mass index, MET; metabolic equivalent; SF-36, short form 36-item health survey; KSQ, Karolinska Sleep Questionnaire; HRV, heart rate variability; LF/HF, ratio between low- and high-frequency spectral powers of HRV.
